# Americans Still Overestimate Social Class Mobility: A Pre-Registered Self-Replication

**DOI:** 10.3389/fpsyg.2015.01709

**Published:** 2015-11-09

**Authors:** Michael W. Kraus

**Affiliations:** Yale School of Management, Yale University, New HavenCT, USA

**Keywords:** social class, replication, intergroup relations, social cognition, motivated cognition

## Abstract

Kraus and Tan (2015) hypothesized that Americans tend to overestimate social class mobility in society, and do so because they seek to protect the self. This paper reports a pre-registered exact replication of Study 3 from this original paper and finds, consistent with the original study, that Americans substantially overestimate social class mobility, that people provide greater overestimates when made while thinking of similar others, and that high perceived social class is related to greater overestimates. The current results provide additional evidence consistent with the idea that people overestimate class mobility to protect their beliefs in the promise of equality of opportunity. Discussion considers the utility of pre-registered self-replications as one tool for encouraging replication efforts and assessing the robustness of effect sizes.

## Introduction

In their original study Kraus and Tan (2015, p. 101) assert that Americans have a widespread belief in the American Dream, that people from “any sector of society, have an equal opportunity to become better educated, earn more money, and obtain whatever job they desire." These widespread beliefs lead to a willful lack of awareness of the actual levels of economic mobility in society. Individuals adhere to exaggerated mobility beliefs for two reasons according to the study: (1) People have little knowledge of statistical information about actual mobility trends; and (2) People are motivated to satisfy basic need to live within a society whose structure is fair and merit-based. To support this prediction, the original paper presented four studies showing that people make large overestimates of mobility in education and income in comparison to available estimates of actual mobility. Moreover, the studies found that these overestimates are largest when made while considering people similar to the self—suggesting these estimates are driven by motivations to protect the self, and that those highest in perceived social class provided the largest overestimates of class mobility—since their elevated status is more meaningful in a hierarchy where mobility is possible (Kraus and Tan, 2015).

In the manuscript, Kraus and Tan (2015, p. 101) make several arguments suggesting the potential practical significance of their research: in particular, they suggest that meaningful public support for economic policy might hinge on “the extent that Americans recognize, and are aware of, the levels of actual social class mobility in society.” The findings from the studies have been reported already in several news websites (Jacobs, 2015; Jaffe, 2015), and in an opinion article for the New York Times (Kraus et al., 2015). The phenomenon of class mobility perceptions has recently been independently investigated by several laboratories, albeit each using slightly different methodologies (Chambers et al., 2015; Davidai and Gilovich, 2015).

In light of the potential practical significance and theoretical importance of the phenomenon in question, we attempted a single pre-registered replication of Study 3 of the original Kraus and Tan (2015) manuscript. We undertook this effort using a roughly identical online sample of workers from Amazon’s Mechanical Turk (*n* = 747), and identical methods as the original study. We chose, in particular, to replicate Study 3 because it allows for a test of all the central predictions of the original manuscript—that Americans overestimate social class mobility, do so to protect the self, and are particularly likely to do so the higher they stand in perceived social class. Importantly, this pre-registered replication attempt follows many of the guidelines outlined in the replication recipe in that it was designed with a careful comparison between the original and replication effects, follows the procedures of the original paper precisely, has high statistical power, and makes the data and analyses publically available (Brandt et al., 2014). Moreover, as an original author on the Kraus and Tan (2015) paper is conducting this replication, a lack of experience and knowledge of the methods and skills used in the first study cannot be an explanation for any discrepancies between the original and replication (Cesario, 2014; Roberts, 2015). In addition, given that the design, analysis, data, and materials used for this replication attempt are pre-registered, the design is robust to any motivated attempts on the part of the author to replicate the original finding at all costs.

Importantly, this pre-registered replication attempt allows us to practically assess whether a number of statistical techniques for detecting publication bias (i.e., r-index, test of insufficient variance, p-curve; Schimmack, 2014a,b; Simonsohn et al., 2014) can be used as predictors of success or failure of an individual high-powered replication attempt. Examination of the original manuscript was conducted using the p-checker application^1^ For the Kraus and Tan (2015) paper, the results show low indices of publication bias: The r-index for the studies was 0.77, indicating the success rate of rejecting the null hypothesis (90%) was close to the observed power in the studies (83.5%; Schimmack, 2014a). The test of insufficient variance, which assesses the extent that the variance of effect sizes matches with expected variance due to sampling error was 2.05, indicating sufficient variance consistent with low publication bias (Schimmack, 2014b). Finally, a p-curve analysis of the statistical tests reported in the paper reveals a pattern of findings consistent with high evidential value *Z* = -15.10, *p* < 0.0001 (go to^2^ for the p-curve and disclosure table; Simonsohn et al., 2014). Together, these publication bias analyses suggest that a high-powered replication attempt of one of the original studies from Kraus and Tan (2015) is likely to deliver similar findings to that of the original manuscript.

In the replication, posted on Open Science Framework^3^, we had participants estimate class mobility on the same six items as in the original study in general, and also with respect to individuals “similar to you in terms of goals, abilities, talents, and motivations.” The latter measure was used to examine how self-relevance influences estimates of class mobility. After these measures, participants estimated statistical information unrelated to class mobility, rated their own knowledge about social class mobility, and filled out demographic information related to perceived social class, income, educational attainment, age, and political ideology. These measures were exactly identical to those used in the original Kraus and Tan (2015) paper.

## Materials and Methods

Methods for this study were pre-registered prior to data collection at Open Science Framework^4^ and made public prior to data collection. The study was approved by the institutional review boards at the University of Illinois and Yale University, and all participants provided consent before completing the experiment. All survey methods and statistical analyses were identical to those reported in Kraus and Tan (2015). We set a target sample size of 700 because such a sample provides more than 95% statistical power to detect an effect size of *r* = 0.14, which was the original Study 3 association between perceived social class and overestimates of class mobility (Kraus and Tan, 2015). The sample of online participants was collected from Mechanical Turk, just as in the original study, returning a sample of more than 700 participants (*n* = 763). Participants were 18 years of age or older (*M* = 33.07, *SD* = 10.88) and were all American citizens. Participants were paid $1.00 for participating in the survey as in the original study. The survey took participants less than 10 min to complete. All participants surveyed were included in analyses except in specific cases where they had missing data (3.88% of all responses).

Participants next completed the same 6-item measure of social class mobility as in Study 3 of Kraus and Tan (2015) in general and with respect to someone who is “similar in terms of goals, abilities, talents, and motivations.” The general (α = 0.63) and the self-relevant estimates (α = 0.60) showed internal consistency at similar levels to the original study (see **Table 1** for correlations between specific mobility estimate items). We computed two mobility estimate composites for general and self-relevant mobility estimates (*r* = 0.79, *p* < 0.0001)—with a score of zero indicating perfect agreement between estimates and actual social class mobility assessed from data in the Current Population Survey (Mishel et al., 2012). Participants then filled out non-mobility estimates for the number of foreign-born 2014 US World Cup soccer players, the number of astronauts who were also military personnel, and the number of astronauts who were also women—scores of zero indicate perfect agreement with statistics on soccer and astronaut demographics^5^,^6^ Lastly, participants filled out demographic information about their perceived social class, annual family income, educational attainment, political ideology, age, and self-rated knowledge about class mobility. All measures were identical to Kraus and Tan (2015).

**Table 1:** Correlations between individual estimates of social class mobility where higher scores indicate greater overestimates of social class mobility.

	Work hours	Education mobility	Students from top income	Students from bottom income	Upward income mobility	Downward income mobility
Work hours	___					
Education mobility	0.48^∗^	___				
Students from top income	0.10^∗^	0.09^∗^	___			
Students from bottom income	0.09^∗^	0.05	0.60^∗^	___		
Upward income mobility	0.37^∗^	0.20^∗^	0.21^∗^	0.34^∗^	___	
Downward income mobility	0.24^∗^	0.14^∗^	0.09^∗^	0.17^∗^	0.45^∗^	___


*^∗^*p* < 0.05.*

## Results

The original study found that participants significantly overestimated social class mobility relative to data on actual mobility trends, observing a large effect (*Cohen’s d* = 3.05). The replication found a similar pattern with respect to general mobility estimates *t*(762) = 36.25, *p* < 0.01, observing a similarly large effect size (*d* = 2.63).

**FIGURE 1:**
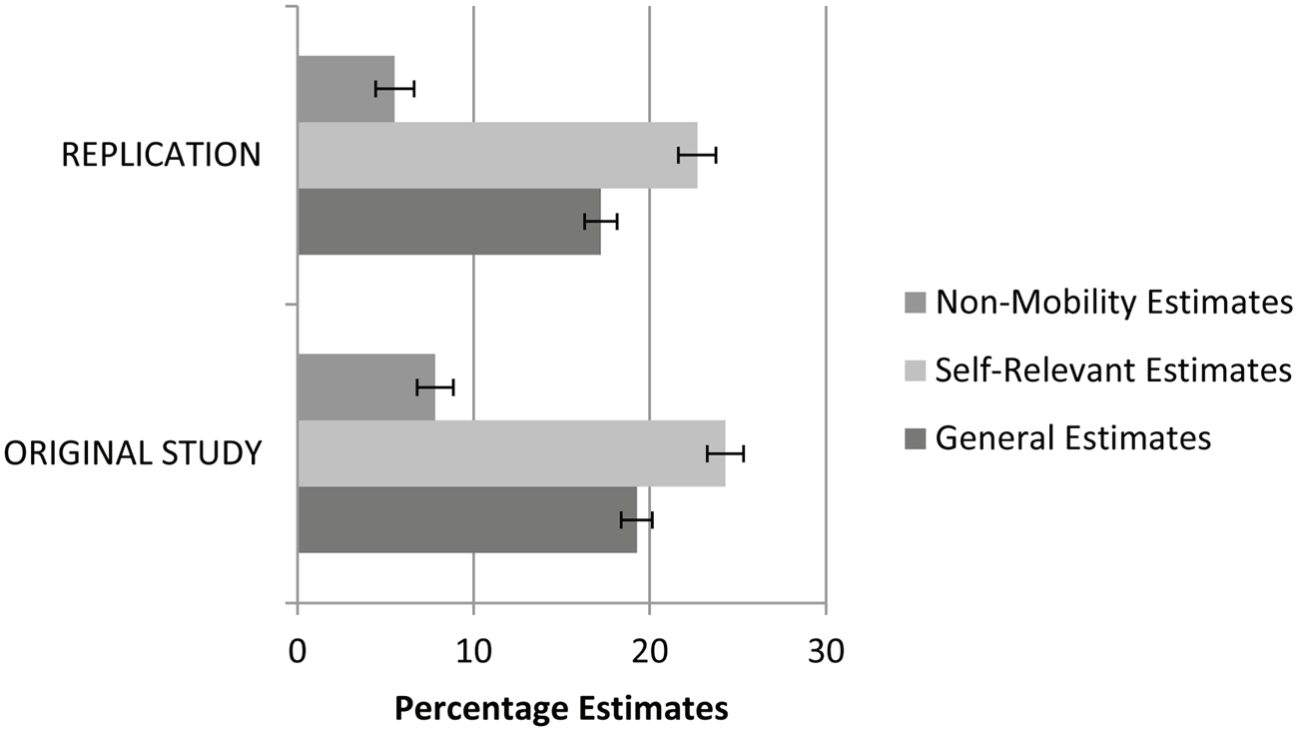
**Estimates of statistical information unrelated to mobility, for class mobility for similar individuals to the self, and for class mobility in general.** A score of zero is equivalent to an accurate estimate. Error bars indicate 95% confidence intervals.

The second prediction from the original study asserts that overestimates of class mobility are more extreme than general estimates of statistical information, and should be even more extreme when made with respect to the self. For this analysis, we conducted a repeated measures analysis of variance (ANOVA) with non-mobility estimates, general class mobility estimates, and self-relevant class mobility estimates as three levels of a single factor. The overall analysis was significant *F*(2,1462) = 316.21, *p* < 0.01, and revealed significant differences between all three groups that aligned with the results from the original study (see **Figure 1**). Specifically, though participants overestimated non-mobility statistics (*M* = 7.32, *SD* = 14.93) they provided larger overestimates of social class mobility in general (*M* = 17.22, *SD* = 12.62), *t*(731) = 14.19, *p* < 0.01, *d*_RM_ = 0.53 (Morris and DeShon, 2002; Lakens, 2013). In comparison to general mobility estimates, participants provided even larger overestimates when thinking about similar others (*M* = 22.70, *SD* = 14.72), *t*(762) = 16.46, *p* < 0.01, *d*_RM_ = 0.62.

We next explored associations between age, social class measures, and class mobility estimates. See **Table 2** for correlational analyses examining associations with general (below the diagonal) and self-relevant (above the diagonal) estimates of class mobility. The observed correlations were similar to those in the prior studies except that self-rated knowledge of social class mobility was not significantly associated with knowledge of mobility in this sample (original *r* = -0.09, *p* = 0.02). As in the original study, conservative ideology and younger age were both moderately associated with greater overestimates of class mobility.

**Table 2:** Correlations between general (below the diagonal) and self-relevant (above the diagonal) social class mobility estimates, social class, age, self-rated mobility knowledge, and political orientation.

	Mobility (Over) estimate	Subjective social class	Age	Education	Income	Knowledge	Political ideology
Mobility (Over) estimate	-	0.24ˆ*	-0.17ˆ*	-0.04	0.12ˆ*	0.02	-0.23ˆ*
Subjective social class	0.23ˆ*	-					
Age	-0.14ˆ*	-0.01	-				
Education	-0.05	0.29ˆ*	0.10ˆ*	-			
Income	0.10ˆ*	0.56ˆ*	-0.06	0.17ˆ*	-		
Knowledge	0.01	0.15ˆ*	0.05	0.12ˆ*	0.04	-	
Political ideology	-0.22ˆ*	-0.12ˆ*	-0.10ˆ*	0.08ˆ*	-0.07ˆ*	0.15ˆ*	-


*^∗^*p* < 0.05.*

**FIGURE 2:**
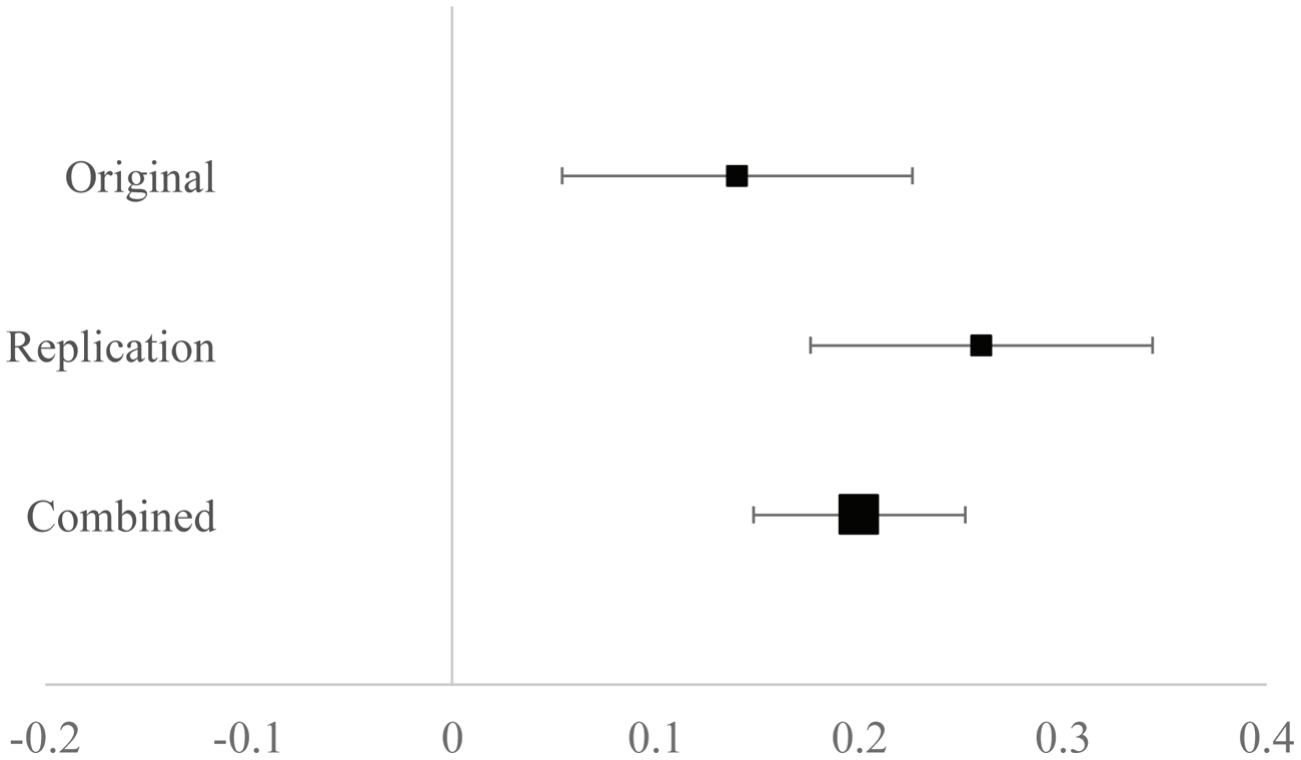
**A forest plot of the association between perceived social class and mobility overestimates after accounting for family income, educational attainment, self-rated knowledge of mobility, age, and political ideology.** Numbers indicate standardized betas for the original study, the replication, and from the combined sample. Error bars indicate 95% confidence intervals. Microsoft excel was used for this plot^7^.

In the original study, perceived social class predicted overestimates of social class mobility even while controlling for age, objective social class measures, political ideology and self-rated knowledge. In the replication a similar pattern emerged: In the full model accounting for self-rated knowledge β = 0.02, *t*(694)* = * 0.59, *p* = 0.55, political ideology β = -0.20, *t*(694) = -5.53, *p* < 0.01, age β = -0.15, *t*(694) = -4.14, *p* < 0.01, income β = -0.06, *t*(694) = -1.35*, p* = 0.18, and education β = -0.09, *t*(694) = 2.27, *p* = 0.02, subjective social class positively predicted mobility overestimates β = 0.26, *t*(694)* = * 5.76, *p* < 0.01. As in the original study regression analysis, younger and more conservative participants tended to provide greater overestimates of class mobility. As **Figure 2** shows, this estimate was slightly larger in magnitude than the estimate from the original study. An estimate of the relationship between perceived social class and mobility estimates was computed based on the combined samples, and is also depicted in **Figure 2.** In the combined sample an effect of perceived social class on mobility estimates was also observed, β = 0.20, *t*(1394) = 6.38, *p* < 0.01^7^

## Discussion

This study tested predictions that Americans tend to overestimate social class mobility in society, tend to do so to protect the self, and tend to provide less accurate estimates the higher they are in perceived social class. The results from this pre-registered exact replication of Study 3 from Kraus and Tan (2015) provide evidence consistent with each of these three predictions: Participants provided large overestimates of social class mobility that became even larger when considered for individuals similar to the self. As well, those higher in perceived social class reported larger overestimates of class mobility than their lower perceived social class counterparts even after accounting for objective measures of social class, self-rated knowledge about class mobility, age, and political ideology. Interestingly, the replication effort produced a larger association between perceived social class and estimates of social class mobility than the original study. Because the studies were exact in design, sampling error is the likely cause of this discrepancy.

Researchers, journalists, and policy-makers are concerned about the replicability of findings in the scientific literature broadly (Estes, 2012; Rolston, 2015), as well as in the social psychology literature in particular (Brandt et al., 2014). As several recent replication initiatives attest (Klein et al., 2014; Nosek and Lakens, 2014), close replications of existing studies happen less frequently than do conceptual replications in social psychology and scientific incentives currently exist to favor conceptual replications over exact ones even at the expense of accuracy. This exact replication attempt was undertaken as part of the increasing focus of social psychology to understand the robustness and consistency of study results reported in the literature.

It is important to note here that statistical assessments of publication bias (e.g., p-curve) suggested the high probability of replication of the original study. These findings suggest the utility of assessments of publication bias like p-curve, r-index, and test of insufficient variance for finding results in social-personality psychology with a high probability of replication. Given that the study was chosen for self-replication knowing the results of these publication bias statistical assessments, a reader would be mistaken to think of these replication findings as somehow indicative of the replicability of the original authors of the manuscript, or of social psychology more broadly. Instead, the results of this work suggest that tools for publication bias do what they are designed for—they can be used as tools to determine the robustness of specific effects within the published literature (e.g., Simonsohn et al., 2014).

Notably, this was a self-replication—a pre-registered replication of one’s own past research. Recently, scholars have called for self-replications because they allow for researchers to conduct studies using their own extensive expertise, thereby contributing to the exactness of experimental methodology (Cesario, 2014; Roberts, 2015). As some criticisms of close replications contend that the researchers who conduct such studies lack experience with the studied methods, self-replications do not suffer from this concern (Schnall, 2014). Moreover, study pre-registration—wherein a time-stamped copy of the study materials, hypotheses, and procedures is posted publically online—prevents researchers from engaging in questionable research practices in order to replicate their own effects at all costs (Roberts, 2015). Thus, any differences between the original studies and self-replications cannot be explained by differences in research expertise or implicit knowledge of experimental procedures. Although there is no substitute for cross validation of effects through replication attempts made by independent laboratories (Klein et al., 2014), self-replications might be an important step towards inviting more close replication, in general, into psychological science.

## Author Contributions

MK designed the study, collected the data and analyzed it, and wrote the manuscript.

## Conflict of Interest Statement

The author declares that the research was conducted in the absence of any commercial or financial relationships that could be construed as a potential conflict of interest.

## Footnotes

1 http://shinyapps.org/apps/p-checker/.

2 https://osf.io/v2bzw/

3 http://osf.io/ecavg

4 https://osf.io/tbw3n/

5 www.espn.com

6 www.nasa.gov.

7 http://mjbrandt.com/2014/06/26/forest-plots-in-excel/.
